# Guillain-Barré Syndrome in an Immunocompromised Patient: A Case Report

**DOI:** 10.7759/cureus.54380

**Published:** 2024-02-17

**Authors:** Enrique Piña-Rosales, Jimena Gonzalez-Salido, Natalia M Barron-Cervantes, Jimena Colado-Martinez, Raúl A Garcia- Santos, Luis Guevara- Arnal

**Affiliations:** 1 Internal Medicine, Fundación Clínica Médica Sur (South Medical Clinical Foundation), Mexico City, MEX; 2 Medicine, School of Medicine, Universidad La Salle (La Salle University), Mexico City, MEX; 3 Medicine, School of Medicine, Universidad Panamericana (Panamerican University), Mexico City, MEX; 4 Neurology, Fundación Clínica Médica Sur (South Medical Clinical Foundation), Mexico City, MEX; 5 Nephrology, Fundación Clínica Médica Sur (South Medical Clinical Foundation), Mexico City, MEX

**Keywords:** polyradiculopathy, viral testing, kidney transplant, organ transplant, guillain-barre syndrome

## Abstract

Guillain-Barré syndrome (GBS) in post-transplant patients is a rare clinical presentation. Although in the literature this neurological condition has been mainly associated with viral infections secondary to immunosuppression, GBS should not only be suspected in patients with an acute condition. It is essential to always rule out a viral or bacterial cause, looking for the most common sources, i.e., urinary, respiratory, and gastrointestinal. The diagnosis of GBS is clinically based, and its management is based on the use of intravenous immunoglobulin (IVIG) or plasma exchange. Its timely diagnosis allows treatment to be started early, thus improving the prognosis of these patients and reducing the time of hospitalization and complications associated with it. This report shows how an interdisciplinary approach is vital in such cases, as both the precipitant and the disease must be managed to decrease the morbidity and mortality associated with this condition. It is crucial to evaluate the benefits and risks of withdrawing immunosuppressive treatment in post-transplant patients, and being able to recognize when restarting them is indicated.

## Introduction

Guillain-Barré syndrome is a group of diseases characterized by acute immune-mediated inflammatory polyradiculoneuropathy. Typically, it consists of a progressive, symmetrical, and ascending paralysis of two or all four limbs, associated with areflexia or hyporeflexia. Sensory symptoms like pain, paresthesia, or hypoesthesia may or may not be present. It progresses in up to four weeks, usually with the maximum disability during the second week, followed by a plateau phase that can last days to months, and finally a phase of progressive improvement. Around 60% to 80% of patients with GBS regain unassisted ambulation within six months [1,2]. It is estimated that there are around 100,000 new cases of GBS worldwide each year. The incidence in the United States and Europe ranges from 0.81 to 1.91 cases per 100,000 people per year [1]. Moreover, the incidence increases by 20% every 10 years [2]. The mean age of presentation is 51 years, and the incidence among men and women is 0.86 vs. 0.57 cases, respectively, per 100,000 people per year [1].

Guillain-Barré syndrome usually presents after an infectious condition, either gastrointestinal, respiratory, or recent immunizations. Microorganisms related to this condition are *Campylobacter jejuni*, Epstein-Barr virus, HIV, and Sars-Cov-2 virus. The pathophysiology consists of molecular mimicry with these infectious agents, involving T and B lymphocytes, macrophages, and humoral immunity. The consequence of this immunomediated response is characterized by peripheral nerve and nerve root injury, either axonal, demyelinating, or both [1-3]. Concerning organ transplantation, the most common association is viral infection, although drug-related causes have also been proposed [4]. In this report, we present the case of a female kidney transplant recipient diagnosed with GBS to further expand the association of organ transplants with the presentation of GBS and highlight the importance of the underlying etiology.

## Case presentation

A 44-year-old female Latin American patient presented to the ER with dyspnea without the presence of chest pain or palpitations and intolerance to the oral route due to nausea and vomiting. Upon direct interrogation, she reported the onset of the condition one week prior to admission, presenting asthenia, adynamia, and generalized weakness of the four extremities, progressive and ascending, with limitations in carrying out activities of daily living, ambulation, and standing. There were no significant hereditary family history details related to the current condition. Her past medical history includes chronic kidney disease secondary to renal hypoplasia in 2014, which progressed to stage V on the Kidney Disease: Improving Global Outcomes (KDIGO) scale that was managed with hemodialysis for the past three years. Currently, she is on kidney transplant therapy from a deceased donor, performed in January 2023. After a three-month postoperative period, she presented with no complications and no chronic diseases.

At the time of presentation, she was on immunosuppressive treatment, including oral prednisone 15 mg every eight hours, mycophenolate 1 g orally every 12 hours, tacrolimus 3 mg orally every 12 hours, and infectious prophylaxis with oral itraconazole 100 mg every 24 hours, valganciclovir 450 mg every 48 hours, and trimethoprim-sulfamethoxazole 160/800 mg every 24 hours. In February 2023, tacrolimus serum levels were reported at 25 ng/ml. Dose reduction was started, and after a month, serum levels decreased to 12.8 ng/mL.

During her stay at the ER, the patient denied any history of fever but reported chills, tremors, and piloerection on various occasions. In addition, she reported the development of irritative urinary symptoms for the previous three days, for which she received treatment with levofloxacin. The appearance of respiratory, gastrointestinal, and urinary tract diseases was questioned, as were immunizations in the previous three weeks, which were denied. On general examination, a rigid, depressible abdomen was found, painful on deep palpation. In the neurological examination, there was no alteration of mental functions or cranial nerves, hypotonia in all extremities, and weakness of the thoracic limbs (4/5 proximal and 3/5 distal). During the examination of the pelvic limbs, the strength evaluated on the Daniels scale was 2/5 proximal and 1/5 distal. Generalized hyporeflexia in the thoracic limbs and areflexia in the pelvic limbs were noted. Likewise, no sensory alterations were found.

Due to the patient’s clinical condition, the decision was made to order laboratory studies to rule out any biochemical cause that could explain her symptoms. A hyperglycemic hyperosmolar state was documented with glucose levels of 729 mg/dL and no presentation of acute kidney injury (AKI). She received adequate treatment with IV fluids and regular insulin infusions, with subsequent resolution. Blood and urine cultures showed no growth of any microorganisms. However, due to the irritative urinary symptoms, it was decided to start her on empirical management with ertapenem IV for five days for the concomitant suspected urinary tract infection. An abdominal ultrasound reported no alterations in the kidney graft.

In parallel, an evaluation was carried out by the neurology service, and a lumbar puncture was performed. Cerebrospinal fluid (CSF) was reported without abnormalities, with zero leukocytes and 41 g/dL of proteins. Additionally, a biofire polymerase chain reaction (PCR) was performed with negative results. Furthermore, negative auramine/rhodamine staining reported Gram without microorganisms, and negative India ink, as well as growths in negative cultures. With the results of the neurological examination and the ruling out of other pathologies through CSF analysis, GBS was diagnosed since it met the clinical criteria [1]. Therefore, it was decided to start the patient on intravenous immunoglobulin (IVIG). Nerve conduction velocities and electromyography were taken, which showed a motor neuropathic alteration of axonal nature with secondary demyelination in nerves of the four extremities with a predominance of the pelvic extremities, thus it was categorized as an acute motor axonal neuropathy (AMAN) variant. Bilateral tibial nerves had prolonged latencies both distally and proximally. The amplitudes were diminished, morphology was dispersed, and nerve conduction velocities were decreased, with no discernible valuable response for the bilateral peroneal nerve and alterations in F waves (Table 1).

**Table 1:** Nerve conduction velocities and electromyography Bilateral F waves of the median, ulnar, and tibial nerves displayed minimal and mean latencies within normal limits, along with dispersed morphology and decreased persistence percentages. NR: Not registered

Nerve	F-latency
Median left	28.0
Median right	26.9
Ulnar left	NR
Ulnar right	NR
Tibial left	53.1
Tibial right	NR

On the second day of treatment with immunoglobulin, there was no further deterioration in neurological symptoms. By the third day, the patient showed improvement in strength. On the fifth day, recovery to assisted ambulation was achieved. Tacrolimus values were found to be 12.8 ng/ml. Therefore, the immunosuppressive regimen was restarted with prednisone and mycophenolate mofetil, without tacrolimus, due to suspicion of being the cause of GBS. In addition, valganciclovir was restarted prophylactically due to the risk of immunosuppression.

During her hospitalization, she didn’t present any hemodynamic, respiratory, or infectious deterioration. However, during her first week of hospitalization, her hemoglobin decreased, reaching 6.7 g/dL, and her platelets decreased to 103 x 103/uL. Due to this, it was determined that the patient had grade II anemia per the WHO classification. The anemia presented as normocytic, normochromic, and regenerative. Iron, folate, and vitamin B12 levels were normal, and the Coombs test was negative. Leukopenia was subsequently determined along with lymphopenia and neutropenia, with total leukocytes of 2.7 x 103/uL, neutrophils of 2.1 x 103/uL, and lymphocytes of 0.5 x 103/uL. Severe pancytopenia was diagnosed. The correction of all cytopenias was treated with the administration of blood products, colony-stimulating factors, romiplostim, and darbepoetin. Eventually, it was concluded that the final diagnosis was pancytopenia secondary to the immunosuppressive treatment that was just restarted.

As an approach for both pathologies presented by the patient, the diagnostic approach was complemented with a hepatitis viral profile. This was reported as negative for viral hepatitis type A (HAV), type B (HBV), and type C (HCV). Also, a test for HIV appeared negative. Specific antibodies (Ab) for other common viral infections were not positive for IgM, which indicated no acute infection (Table 2).

**Table 2:** Laboratory results of specific antibodies for  common viral infections Ab: Antibody, EBV: Epstein-Barr virus, PCR: Polymerase chain reaction, CMV: Cytomegalovirus

Parameter	Value	Interpretation
Ab vs. Parvovirus B19 IgM	0.2 U/mL	Negative
Ab vs. Parvovirus B19 IgG	5 U/mL	Positive
Ab vs. Epstein-Barr virus (EBV) IgM	11.8 U/mL	Negative
Ab vs. EBV IgG	528 U/mL	Positive
PCR-EBV	Positive	Positive
Ab vs. Cytomegalovirus (CMV) IgM	5.8 U/mL	Negative
Ab vs. CMV IgG	568 U/mL	Positive

Due to the previously mentioned negative results, a biopsy and bone marrow aspirate were performed, and the final histopathological findings of aplastic anemia were reported. The biopsy showed hypocellular bone marrow (10%) with an alteration in the maturation of the three hematopoietic lines, so the aforementioned treatment was continued. By day 10 of hospitalization, the patient had regained strength (4/5) in her four limbs, but with the persistence of generalized hyporeflexia. Subsequently, mycophenolate mofetil was changed to cyclosporine A because of aplastic anemia and hydrocortisone; this was her final immunosuppressive regimen. Due to a favorable neurological evolution and an increase in cell lines, the patient was discharged without incident.

## Discussion

The global incidence of GBS averages 1.1 cases per 100,000 person-years; additionally, in Mexico, 0.79 cases per 100,000 person-years were reported in 2019 [2,4]. Some GBS cases have been reported after solid organ and bone marrow transplants. Among these cases, 13 were linked to the liver, 11 to the kidney, six to the heart, six to the lung, and two to bone marrow, with a greater occurrence among males [5,6]. Given the autoimmunity component in GBS, it may initially appear implausible that it can also be linked to immunosuppressive states. After all, autoimmunity is characterized by an overproduction of antibodies, whereas immunosuppression is marked by a reduced capacity to produce them. Nevertheless, research has demonstrated that GBS is indeed associated with HIV infection, acute leukemia, and even bone marrow transplant recipients taking immunomodulators [4]. Within transplant recipients who later develop GBS, the infection most commonly associated with it is caused by the cytomegalovirus (CMV). It is essential to actively search for this virus due to its ability to remain latent in endothelial cells, smooth muscle cells, fibroblasts, and monocytes. This becomes a priority, particularly in patients with donors who have a positive CMV serology. In our case, CMV IgM serology was negative. It is possible to have a valganciclovir-resistant CMV breakthrough despite prophylaxis; this can be confirmed through the CMV viral load. In this case, it’s important to mention that the CMV viral load wasn’t checked. Moreover, GBS in post-kidney transplant patients is widely recognized to be linked to CMV, as demonstrated by Ostman and Chacko, who showed that CMV was responsible for 80% of the associated infections [7]. 

Some cases of GBS associated with the post-transplant condition have been attributed to immunosuppressive therapy. Sharma et al. reported two cases of lung transplant recipients who developed GBS, and this condition was found to be linked to their tacrolimus therapy [8]. Furthermore, Kaushik et al. described a case of Miller-Fisher syndrome in a post-liver transplant patient, which was associated with the use of tacrolimus [9]. Additionally, Falk et al. reported a GBS case with the administration of cyclosporine [10]. Jakes et al. presented a case of a post-kidney transplant patient with GBS, probably linked to tacrolimus, while Meena et al. and Jakovler et al. reported similar cases in 2020 and 2018, respectively [6,11,12]. Some of the hypotheses proposed to explain tacrolimus-induced polyradiculopathy and other immunosuppressants suggest an inflammatory phenomenon due to their various effects on T lymphocyte subpopulations, including the activation of self-reactive T lymphocyte subpopulations [4,13]. However, it is crucial to rule out pathologies such as mononeuropathy and polyneuropathy induced by tacrolimus. These conditions exhibit neurotoxic effects at increased tacrolimus levels, and neuroconduction studies may indicate demyelination [14].

In the presented case, the ascending and symmetric progression of weakness, findings indicative of non-demyelinating axonal motor neuropathy, the absence of elevated tacrolimus levels, and the response to treatment confirm the suspicion of GBS. Similarly, it is necessary to rule out the possibility of chronic inflammatory demyelinating polyneuropathy (CIDP). The presence of CIDP has been reported infrequently in patients who have undergone solid organ transplants, with an incidence of 0.6% [4,10]. In our case, the likelihood of this pathology is also low, given the rapid onset of weakness, its non-insidious nature, and the absence of other symptoms during follow-up. However, the influence of other medications or unidentified pathogens cannot be entirely ruled out. Regarding treatment, there is no established guideline for management in GBS cases among transplant recipients. In prior cases of kidney and liver transplants, intravenous immunoglobulin administration and plasma exchange have been utilized, yielding satisfactory neurological outcomes along with the discontinuation of the immunosuppressant [4].

## Conclusions

Guillain-Barré syndrome is a rare occurrence in post-transplant patients and is often linked to viral infections in the context of immunosuppression. It is crucial not to limit the diagnostic suspicion to acute conditions, as GBS can manifest in various clinical scenarios. Thorough evaluation for viral or bacterial causes is imperative. This was the first known case of GBS associated with a solid organ transplant at our institution. This underscores its significance and its value as a pioneer in these challenging-to-find cases. Among its limitations, we acknowledge the inherent nature of the study and the lack of historical information regarding the externally performed transplant. Additionally, a urinary tract infection could have been the trigger for GBS; however, it did not have a sufficient duration, suggesting it was an unrelated event. In this case, the trigger of GBS remains unclear. Negative CMV IgM might not be sensitive enough to totally rule out reactivation. Also, with an elevated tacrolimus trough level, it is possible that this is a polyneuropathy due to tacrolimus-induced neurotoxicity. It is crucial to remain vigilant for signs of neurological deterioration in transplant recipients and to rule out potential causes, such as recent infections, adverse effects of medications, or uncommonly mediated immunological phenomena.
